# Long-term treatment of Parkinson’s disease with levodopa and other adjunctive drugs

**DOI:** 10.1007/s00702-016-1671-x

**Published:** 2017-01-16

**Authors:** Yoshikuni Mizuno, Satoe Shimoda, Hideki Origasa

**Affiliations:** 1 Department of Neurology, Juntendo University School of Medicine, Tokyo, Japan; 2 Tokyo Clinic, Tokyo, Japan; 3 Ginza Medical and Neurological Clinic, Tokyo, Japan; 4 Division of Biostatistics and Clinical Epidemiology, University of Toyama Graduate School of Medicine and Pharmaceutical Sciences, Toyama, Japan

**Keywords:** Parkinson’s disease, Long-term treatment, Levodopa, Wearing off, Dyskinesia

## Abstract

We report a long-term treatment of Parkinson’s disease in out-patient clinics. The patients with Parkinson’s disease were evaluated at the time of clinic visit from September 1st, 2015 to February 29th, 2016. Total number of the patients was 498. The age at the evaluation was 69.9 ± 9.3 years and the age of onset was 60.2 ± 11.3. Hoehn and Yahr severity was 3.28 ± 0.94 in patients who were from 16 to 20 years (*n* = 53) and 3.00 ± 0.86 in patients from 21 years or more (*n* = 38) from the onset of the disease to the evaluation. The dose of levodopa was 741 ± 295 mg per day and the number of levodopa dosing was 5.85 ± 2.59 times in 16–20 years from the onset to the evaluation and 703 ± 251 mg/day and 6.03 ± 3.20 times a day in 21 years or more from the onset to the evaluation. Levodopa was given in most cases into an empty stomach. The incidence of wearing off was 73.6% and dyskinesia was 37.7% in the 16–20 years group and 76.3% and 55.3% in 21 years or more group, respectively. The patients who had 15 years or less from the onset to the evaluation had much milder severity of the disease. Hoehn and Yahr severity, the dose of levodopa, and the incidence of wearing off were about the same as in the literature. But the incidence of dyskinesia was much lower than those appeared in the literature. We discussed reasons why the incidence of dyskinesia was lower in our study.

## Introduction

It is not well known about the ideal long-term treatment of Parkinson’s disease (PD). Initial treatment with levodopa is in most of the patients smooth. At the first motor symptom of PD, still approximately 50% of ^18^F-dopa uptake in the striatum is remaining (Brooks 2004). Therefore, levodopa is likely taken up into the remaining dopaminergic neurons, which are equipped with the dopamine transporters. Therefore, there will be no wearing off or dyskinesia. Dopamine once released to the synaptic cleft is rapidly taken up by the dopamine transporter to the original dopaminergic neurons. Thus, dopamine is re-used again when it is necessary.

Then the degeneration of dopaminergic neurons in the substantia nigra progresses slowly in PD despite the treatment with levodopa. Then the patients with PD realize that some worsening of their symptoms before the next dose of levodopa. Then the physicians in charge have to prescribe levodopa four times or more a day. This is the wearing off. In addition, physicians in charge may have to prescribe levodopa when the patients’ stomachs are empty to get a maximum effect of levodopa.

How about the frequencies of wearing off and dyskinesia in the literature? Wearing off fluctuations have been reported to be 40–50% of patients treated with levodopa for 5 years and approximately two-thirds in patients treated for 10 years or more. Dyskinesias have been reported to be 40–50% in patients treated with levodopa for 5–10 years or more (Caraceni et al. 1991; Reardon et al. 1999; Schrag and Quinn 2000; Rascol et al. 2000: Ahlskog and Muenter 2001; Parkinson Study Group 2004; Hauser et al. 2007; Parkinson Study Group CALM Cohort Investigators 2009: Colombo et al. 2015). The longer the treatment period, the more patients would show dyskinesia.

At this stage of levodopa treatment when the wearing off is present, where levodopa would be decarboxylated to dopamine? Levodopa is taken up by the aromatic amino acid uptake system to the cells, i.e., dopamine, noradrenalin, and serotonin neurons but also non-neuronal tissues. Levodopa is still effective although the duration of on period is shortened. In addition, no dopamine transporter can be visualized in the DAT scan in the posterior putamen where the motor loop to the frontal cortex is of upmost importance in voluntary movements. Here levodopa would be decarboxylated to dopamine at least in part in the serotonergic neurons coming from the raphe nucleus in the pons. The serotonergic neurons are equipped with the serotonin transporter but not the dopamine transporter. Therefore, dopamine released from the serotonergic neurons is not taken up through the serotonin transporter. Thus, dopamine released into the synaptic clef will be metabolized by monoamine oxidase and catechol-*O*-methyltransferase. Therefore, dopamine in the serotonergic neurons would not stay long in the basal ganglia. We believe that this is one of the cellular mechanisms for wearing off. When the synaptic cleft dopamine is too much, dyskinesia may result. In recent years, a number of articles suggested the serotonergic neurons for decarboxylation of dopamine (Bara-Jimenez et al. 2005; Cheshire and Williams 2012; Politis et al. 2014; Smith et al. 2015; Cheshire et al. 2015; Lee et al. 2015; Roussakis et al. 2016).

Keep these facts in mind, we treated patients with PD so that the long-term complications of levodopa will become as low as possible.

## Methods

### Style

This is an observational study. The patients with PD were evaluated at the time of clinic visits from September 1st, 2015 to February 29th, 2016.

### Diagnosis of PD

Those patients who fulfilled the following criteria were included, i.e., presence of parkinsonism according to the British Council definition (Hughes et al. 1992), reduced uptake of cardiac meta-iodobenzylguanidine (MIBG), and normal brain MRI other than the aging-related changes. In some patients, cardiac MIBG was not examined; in such cases, response to levodopa was confirmed.

### Drug treatment

Levodopa was used when the patients were 65 years or older. When the patients were less than 60 years, a dopamine agonist was used. When the patients were 60 years or older but not reaching the 65 years, a case by case principle was adopted.

### Dose of levodopa

As an initial dose, we gave to the patients 200–300 mg of levodopa with a decarboxylase inhibitor (DCI) in a day in two to three divided doses after each meal. When this amount did not produce a satisfactory result, levodopa with DCI was given shortly before the meal. Still the amount of levodopa appeared to be not enough, the levodopa dose was increased gradually to 450 mg or to 600 mg a day. If this amount still is not satisfactory to improve patients’ parkinsonian symptoms, other drugs were added (see below). When the patient had wearing off phenomenon, levodopa with DCI was given according to the length of the on period. When the effect of levodopa wears off, we instructed the patients to take the next dose of levodopa right after the symptoms of wearing off. Each dose of levodopa was 50–200 mg in this case. If a patient had non-motor symptoms such as headache, lumbago, or any other symptoms that lead to off state, levodopa with DCI was given at the onset of such non-motor symptoms.

When a patient developed inter-dose dyskinesia, each levodopa dose was decreased when it was possible, and the number of giving levodopa in a day was increased so that the daily dose of levodopa would not change much. Other anti-parkinsonian drugs was reduced or discontinued if possible except for the anti-cholinergics and amantadine HCl. Amantadine HCL was prescribed at times to decrease dyskinesia.

### Other medication

Other medications such as a monoamine oxidase B inhibitor (selegiline), catechol-*O*-methyltransferase inhibitor (entacapone), dopamine agonists (pramipexole, ropinirole, rotigotine, or pergolide), trihexyphenidyl, amantadine HCl, zonisamide, istradefylline, and/or dops were used when indicated. Entacapone (100 mg at each time) was used at the same time with levodopa. When levodopa was given nine times or more in a day, the last dose was given without entacapone.

### Treatment of non-motor symptoms

Constipation, nocturia, hypotension, pre-tibial pitting edema, pain, sleep disorders, excessive daytime sleepiness, anxiety state, depression, fatigue, impulse control disorders, dopamine dysregulation syndromes, hallucinations, delusions, psychosis, and dementia were treated as possible with appropriate drugs.

### Semi-quantitative evaluation

Hoehn and Yahr severity of the disease was evaluated when the patients were on. Resting tremor, rigidity, and bradykinesia were semi-quantitatively evaluated according to the UPDRS. Gait disturbance was evaluated according to the following criteria; 0, normal; 1, mild gait disturbance; 2, moderated gait disturbance; the patient would not fall when he or she walks alone, 3, marked gait disturbance; the patient would fall when he or she walks alone, 4, unable to walk. Retropulsion was evaluated according to the following criteria: 0, no retropulsion; 1, mild retropulsion; the patient would recover and he or she would not fall; 2, moderate retropulsion, the patient would fall when he or she is not supported; 3, marked retropulsion, the patient would fall when he or she stands alone; 4, unable to stand because of unsteadiness of standing. Wearing off was evaluated according to the following criteria; 0, no wearing off; 1, mild wearing off; taking levodopa four time or less in a day because of wearing off, 2, moderated wearing off; taking levodopa 5–7 times in a day, 3, marked wearing off; taking levodopa 8–10 times in a day, 4, severe wearing off; taking levodopa 11 times or more in a day. Dyskinesia was evaluated according to the following criteria; 0, no dyskinesia; 1, mild dyskinesia; the patient may not realize the presence of dyskinesia, 2, moderate dyskinesia, the patient realizes the dyskinesia; but not bothered by the dyskinesia in the daily life, 3, marked dyskinesia, the patient realizes the dyskinesia and bothered by the dyskinesia in the daily life; 4, severe dyskinesia, the patient feels fatigue and/or difficulty in the daily life because of dyskinesia. Freezing was evaluated according to the following criteria: 0, no freezing; 1, mild freezing; the freezing occurs at home or outside of the home, but not in the examining room; 2, moderate freezing; the freezing occurs at home and/or outside of the home, and in the examining room. Hallucination was evaluated according to the following criteria; 0, none; 1, mild hallucination; the patient realizes that it is the hallucination but not bothered from it, 2, moderate hallucination; hallucinations occur mainly during night, patients realize that it is hallucination and sometime embarrassing and annoying. 3, marked hallucination; hallucinations occur day and night, patients may not realize that it is the hallucination. 4, severe hallucination; the patient does not realize that it is the hallucination and the patient may talk to the hallucination; the patient may lose orientation or may be excited. Dementia was evaluated according to the following criteria, 0, no dementia; 1, mild dementia; dementia does not need observation and it does not interfere with the daily life; 2, moderate dementia; dementia may need observation, 3, marked dementia; dementia needs observation, but he or she is oriented to the outline of the home and its vicinity; 4, severe dementia, dementia needs observation; he or she is disoriented to the home and its vicinity.

### Statistical analysis

Values represent mean and standard deviation where Hoehn and Yahr severity was transformed into integer score. Mean age at onset was compared between male and female by Student’s *t* test. Average score of Hoehn and Yahr severity was compared by Wilcoxon rank-sum test.

## Results

### Numbers of patients

The total number of the patients was 498 (male 222, female 276).

### The age at evaluation and the age of onset

The time intervals from the onset of the disease and the time of the evaluation were divided into 5 groups, i.e., the interval between the onset and the evaluation 5 years or less, 6–10 years, 11–15 years, 16–20 years, and 21 years or more (Table 1). The age at the evaluation was 69–71 in average in all groups. The ages at the onset were gradually decreased as the observation period increased.

**Table 1:** The number of patients, male/female, the age, the age at the onset, and the duration from the onset

From the onset	Number	Male/female	Age	Age at onset	Duration from onset (Years)
5 years or less	135	61/74	69.2 ± 10.0	66.2 ± 10.0	3.49 ± 1.30
6–10 years	158	61/97	69.7 ± 9.1	62.6 ± 9.2	7.86 ± 1.37
11–15 years	114	56/58	70.3 ± 8.3	58.4 ± 8.4	12.43 ± 1.27
16–20 years	53	26/27	70.9 ± 9.2	53.8 ± 8.9	17.58 ± 1.51
21 years or more	38	18/20	70.7 ± 9.5	43.4 ± 12.7	27.79 ± 6.84
Total	498	222/276	69.9 ± 9.3	60.2 ± 11.3	10.28 ± 7.05

Mean ± standard deviation

There was no significant difference between the male and the female in the age and the age of onset. The age at the evaluation was 70.0 ± 9.5 years in males, 69.9 ± 9.1 years in females (ns), and 69.9 ± 9.3 years in total. The age of onset was 58.9 ± 12.3 years in males, 60.5 ± 10.5 years in females (non-significant), and 60.2 ± 11.3 years in total.

### Symptoms at the age of onset

Symptoms at the onset were tremor in 49.8%, gait disturbance in 33.3%, and symptoms related to bradykinesia in 16.9%. Bilateral involvement with tremor at the onset was rare. In the remaining, those patients who had gait disturbance at the onset had unilateral or bilateral involvement, and those who had symptoms related to bradykinesia had usually unilateral hand involvement.

### Hoehn and Yahr severity

Hoehn and Yahr severity of the patients who had the tremor at the onset (2.52 ± 1.01) was less than the patients who had gait disturbance or bradykinesia at the onset (2.80 ± 0.96) (*p* < 0.0001). There was no significant difference in the age and the age of onset between these two groups. Hoehn and Yahr severity according to the breakdown of the intervals between the age of onset and the age of the evaluation is shown in Table 2. Hoehn and Yahr severity increased from the group of 5 years or less to the group of 16–20 years from the onset to the evaluation (3.28 ± 0.94). But it was less than that in the group of 21 years or more (3.00 ± 0.86). This was in part due to the presence of early onset PD with good prognoses in the group of 21 years or more.

**Table 2:** The Hoehn and Yahr severity

From the onset	No.	Hoehn and Yahr	0 degree		1st degree		2nd degree		3rd degree		4th degree		5th degree		3rd or less	
5 years or less	135	2.24 ± 0.98	10	7.4%	6	4.4%	75	55.6%	31	23.0%	11	8.1%	2	1.5%	122	90.4%
6–10 years	158	2.54 ± 0.94	6	3.8%	2	1.3%	78	49.4%	46	29.1%	24	15.2%	2	1.3%	132	83.5%
11–15 years	114	2.91 ± 0.88	0	0.0%	0	0.0%	47	41.2%	33	28.9%	31	27.2%	3	2.6%	80	70.2%
16–20 years	53	3.28 ± 0.94	0	0.0%	0	0.0%	12	22.6%	20	37.7%	15	28.3%	6	11.3%	32	60.4%
21 years or more	38	3.00 ± 0.86	0	0.0%	0	0.0%	12	31.6%	16	42.1%	8	21.1%	2	5.3%	28	73.7%
Total	498	2.66 ± 0.99	16	3.2%	8	1.6%	224	45.0%	146	29.3%	89	17.9%	15	3.0%	394	79.1%

Mean ± standard deviation

### Dose of levodopa

Doses of levodopa are shown in Table 3. It increased from the group of 5 years or less from the onset to the group of 16–20 years from the onset (416 ± 223 to 741 ± 295 mg a day). It was somewhat less in the group of 21 years or more from the onset (703 ± 251 mg). Numbers of levodopa doses increased from the group of 5 years or less to the group of 21 years or more (3.29 ± 1.52 to 6.03 ± 3.20).

**Table 3:** Levodopa dose, number of dosing, ranging of dosing, and dose per day

From the onset	No.	On levodopa	Dose	No of dosing	Range of dosing	Dose per day (mg)
5 years of less	135	119 (88.1%)	416 ± 223	3.29 ± 1.52	0–8	0–800
6–10 years	158	157 (99.4%)	614 ± 256	4.99 ± 2.69	0–20	0–1200
11–15 years	114	113 (99.1%)	731 ± 285	5.43 ± 2.39	0–12	0–1500
16–20 years	53	53 (100%)	741 ± 295	5.85 ± 2.59	3–12	200–1500
21 years of more	38	38 (100%)	703 ± 251	6.03 ± 3.20	2–16	200–1500
Total	498	480 (96.4%)	613 ± 297	4.85 ± 2.64	0–20	0–1500

*Dose* mean ± standard deviation in those who were on levodopa

### Other anti-PD drugs

Other anti-PD drugs, which were taken by the patients at the time of the evaluations, are listed in Table 4. Dopamine agonists and trihexyphenidyl were the two most concomitant drugs with levodopa. They were used in approximately half of the patients.

**Table 4:** Anti-PD drugs other than levodopa; the number of patients who were taking respective drugs

From the onset	No.	DA agonists		Trihexyphenidyl		Amantadine		Selegiline		Entacapone		Zonisamide		Istradefylline		L-dops	
5 years or less	135	34	25.2%	57	42.2%	10	6.3%	9	6.7%	5	3.7%	10	7.4%	0	0.0%	0	0.0%
6–10 years	158	76	48.1%	72	45.6%	16	10.1%	22	13.9%	13	8.2%	19	12.0%	3	1.9%	0	0.0%
11–15 years	114	76	48.1%	54	47.4%	27	23.7%	13	11.4%	19	16.7%	16	14.0%	4	3.5%	1	0.9%
16–20 years	53	32	60.4%	23	43.4%	11	20.8%	6	11.3%	12	22.6%	11	20.8%	2	3.8%	1	1.9%
21 years or more	38	21	55.3%	18	47.4%	19	50.0%	1	2.6%	8	21.1%	8	21.1%	1	2.6%	0	0.0%
Total	498	223	44.8%	224	45.0%	83	16.7%	51	10.2%	57	11.4%	64	12.9%	10	2.0%	2	0.4%

### Tremor, rigidity, bradykinesia, gait disturbance, and retropulsion

The numbers of patients who had these symptoms at the time of the evaluation are listed in Table 5. At the time of onset, tremor was present in about 50% of the patients. But it reduced to 23.0% to the group of 5 years or less and further reduced in the remaining groups to approximately 15%. The overall presence of tremor was 16.1%.

**Table 5:** Tremor, rigidity, bradykinesia, gait disturbance, and retropulsion

From the onset	No.	Levodopa	Hoehn and Yahr	Tremor		Rigidity		Bradykinesia		Gait disturbance		Retropulsion	
5 years or less	135	366 ± 223	2.24 ± 0.98	31	23.0%	49	36.3%	106	78.5%	95	70.4%	33	24.4%
6–10 years	158	610 ± 256	2.54 ± 0.94	21	13.3%	54	34.2%	127	80.4%	124	78.5%	45	28.5%
11–15 years	114	731 ± 285	2.91 ± 0.88	13	11.4%	62	54.4%	104	91.2%	97	85.1%	46	40.4%
16–20 years	53	741 ± 295	3.28 ± 0.94	9	17.0%	27	50.9%	49	92.5%	48	90.6%	28	52.8%
21 years or more	38	703 ± 251	3.00 ± 0.86	6	15.8%	15	39.5%	35	92.2%	36	94.7%	14	36.8%
Total	498	613 ± 297	2.66 ± 0.99	80	16.1%	207	41.6%	421	84.5%	400	80.3%	166	33.3%

Levodopa dose and Hoehn and Yahr indicate mean ± standard deviation in all the patients in respective classes

Rigidity remained in 41.6% of the patients, but in many of the patients, it was usually present in the neck and usually milder or none in the extremities. Bradykinesia remained in 84.5% and gait disturbance in 80.3% of the patients. These were the two major symptoms, which remained in most of the patients. Retropulsion was remaining in 33.3% of the patients. In many cases, retropulsion was present initially but it tended to disappear in subsequent years.

### Wearing off, dyskinesia, freezing, hallucination, and dementia

Wearing off was only 17.8% in the group of 5 years or less, but it increased to 56.3% in the group of 11–15 years; it reached approximately 75% thereafter (Table 6). Dyskinesia was noted only in 2.2% in the group of 5 years or less from the onset. It was still 17.7% in the group of 6–10 years. In the group of 11–15 years, it increased to 31.6%, and in the group of 16–20 years, to 37.7%. In the group of 21 years or more, it was 55.3%, apparently in part due to the increase of early onset patients. Freezing increased from 30.4% in the group of 5 years or less to 76.3% in the group of 21 years or more. Hallucinations (mainly visual) were not often (5.6%). Apparently, this was mainly due to the treatment effects: those patients who had hallucinations were treated by donepezil or quetiapine or both, and in many patients hallucinations disappeared at the time of the evaluation. Dementia was noted in 20.5% as the total. It was highest in the group of 16–20 years from the onset to the evaluation (39.6%).

**Table 6:** Wearing off, dyskinesia, freezing, hallucination, and dementia

From the onset	No.	Wearing off		Dyskinesia		Freezing		Hallucination		Dementia	
5 years or less	135	24	17.8%	3	2.2%	41	30.4%	5	3.7%	16	11.9%
6–10 years	158	89	56.3%	28	17.7%	88	55.7%	7	4.4%	22	13.9%
11–15 years	114	86	75.4%	36	31.6%	67	58.8%	5	4.4%	35	30.7%
16–20 years	53	39	73.6%	20	37.7%	35	66.0%	7	13.2%	21	39.6%
21 years or more	38	29	76.3%	21	55.3%	29	76.3%	4	10.5%	8	21.1%
Total	498	267	53.6%	108	21.7%	260	52.2%	28	5.6%	102	20.5%

Data indicate the number of patients who were positive in respective symptoms and the percentage

### Hoehn and Yahr severity, bradykinesia, and gait disturbance according to the age of onset

These are listed in Table 7. Patients with the age of onset 70 years or older had shortest period from the onset of disease to time of the evaluation. Despite that, these patients marked the worst value of the Hoehn and Yahr at the time of the evaluation. Bradykinesia and gait disturbance scores were the worst two in this group.

**Table 7:** Five major symptoms according to the age of onset

Age of onset	No.	Levodopa	Hoehn and Yahr	Tremor		Rigidity		Bradykinesia		Gait disturbance		Retropulsion	
39 or younger	22	573 ± 291	2.32 ± 0.70	7	31.8%	9	40.9%	17	77.3%	17	77.3%	3	13.6%
40–49 years	52	679 ± 311	2.40 ± 0.63	6	11.5%	20	38.5%	41	78.8%	37	71.1%	8	15.4%
50–59 years	147	618 ± 319	2.56 ± 1.00	17	11.6%	67	45.6%	122	83.0%	113	76.9%	44	29.9%
60–69 years	172	592 ± 291	2.66 ± 1.05	34	19.8%	66	38.4%	142	82.6%	136	79.1%	55	32.0%
70 or older	105	509 ± 242	3.00 ± 1.00	16	15.2%	45	42.9%	99	94.3%	97	92.4%	56	53.3%
Total	498	613 ± 297	2.66 ± 0.99	80	16.1%	207	41.6%	421	84.5%	400	80.3%	166	33.3%

Levodopa dose and Hoehn and Yahr indicate mean ± standard deviation in all the patients in respective classes

## Discussion

By reviewing the above results, (1) the overall outcome in the Hoehn and Yahr severity (2.66 ± 0.99, disease duration 10.28 ± 7.05 years) is about the same as reported in the literature (Schrag and Quinn 2000, 2.4 ± 0.8, disease duration 6.8 ± 4.3 years; Nissinen et al. 2009, 2.4 ± 0.6, disease duration 10.9 ± 5.4 years). (2) Levodopa doses (613 ± 297 mg) and the numbers of drug intake in a day are approximately the same as in the literature (Rascol et al. 2000, 753 ± 398 mg; Parkinson Study Group 2004, 702 ± 461 mg; Nissinen et al. 2009, 692 ± 364 mg). (3) Use of trihexyphenidyl is more frequent (45%) than the recent literature but the dose of trihexyphenidyl is much smaller than that in the literature (Parkinson Study Group 2004, 4.7%; Nissinen et al. 2009, 16%). (4) The incidence of wearing off (17.8–76.3%, total mean 53.6% in 10.28 ± 7.05 years) is about the same as in the literature (Caraceni et al. 1991, 48% in 2–10 years; Reardon et al. 1999, 34% in 15 months; Schrag and Quinn 2000, 63% in 10 years or more; Parkinson Study Group 2004, 62.7% in 4 years; Hauser et al. 2007, 72.0% in 10 years; Parkinson Study Group CALM Cohort Investigators 2009, 58.8% in 6 years: Colombo et al. 2015, 63.7% for men in more than 5 years, 73.5% for women in more than 5 years of treatment with levodopa). (5) The frequency of dyskinesia, however, is much less (2.2–55.3%, total mean 21.7% in 10.28 ± 7.05 years) than those in the literature (Reardon et al. 1999, 53% in 15 months; Schrag and Quinn 2000, 53% in 10 years or more; Rascol et al. 2000, 45% in 5 years; Ahlskog and Muenter 2001, 40% in 4–6 years; Parkinson Study Group 2004, 54.0% in 4 years; Hauser et al. 2007, 77.8% in 10 years; Parkinson Study Group CALM Cohort Investigators 2009, 36.8% in 6 years of treatment with levodopa). One report from China reported low incidence of dyskinesia (Chen et al. 2014, 19.3% in 10 years or more of treatment with levodopa).

Frequency of dyskinesia was much lower than those in the literature, which we did not expect when the evaluations were done. In the group of patients who had PD for 16–20 years, the frequency of dyskinesia was 37.7%. As our study is an open study, we did various things when patients started to have dyskinesia such as frequent dosing of levodopa, lower amount of levodopa at each dose, reducing or discontinuing concomitant anti-Parkinson drugs such as a monoamine oxidase B inhibitor, entacapone, dopamine agonists, zonisamide, or istradefylline. We did not reduce amantadine HCl or trihexyphenidyl when used unless patients had hallucinations or cognitive or psychiatric side effects.

Today, the serotonergic neurons in the basal ganglia is claimed to be responsible for levodopa-induced dyskinesia. The earliest literature discussing the relationship between levodopa-induced dyskinesia and the serotonergic system in the striatum is that of Bara-Jimenez et al. (2005). They gave sarizotan, a serotonergic 5-HT1A agonist, to patients with advanced PD with dyskinesias at 2 and 5 mg twice a day to 18 PD patients with dyskinesia. They found that sarizotan co-administration reduced levodopa-induced dyskinesias. They suggested that 5-HT1A agonists might be useful as a levodopa adjuvant in the treatment of PD. Sprouting of the serotonergic neurons to the striatum in MPTP-lesioned mice is well known (Rozas et al. 1998; Lee et al. 2008). In experimental animals, dopa-decarboxylase-immuno-reactive neurons were noted in dopamine-depleted striata (Lopez-Real et al. 2003). Therefore, it seems likely that decarboxylation of levodopa takes place in serotonergic neurons in the striatum in advanced PD (Lopez et al. 2001; Bara-Jimenez et al. 2005; Yamada et al. 2007; Cheshire and Williams 2012; Politis et al. 2014; Smith et al. 2015; Cheshire et al. 2015; Lee et al. 2015). Lopez et al. (2001) observed that extrinsic levodopa given to dopamine denervated rats was still able to induce the rotational behavior and concluded that the serotonergic neurons in the striatum might be responsible for conversion of the exogenous levodopa to dopamine. Yamada et al. (2007) showed extrinsically levodopa-derived dopamine in the serotonergic neurons in the basal ganglia in rats. But these serotonergic neurons do not have dopamine transporters. The serotonin transporters cannot reuptake dopamine once released from the serotonergic neurons into the synaptic clefts (Mortensen et al. 1999; Mantovani et al. 2009). This inability to re-use dopamine would shorten the effect of levodopa, and the amount of dopamine released into synaptic cleft is too much, it would result in peak-dose dyskinesia.

Treatments with the serotonin agonist such as 8-hydroxy-2-(di-*n*-propylamino)-tetralin (8-OH-DPAT) were reported to reduce dyskinesia in experimental animals (Tomiyama et al. 2005; Carta et al. 2007; Muñoz et al. 2009; Nahimi et al. 2012; Iderberg et al. 2015; Paolone et al. 2015; McCreary et al. 2016; Ghiglieri et al. 2016). The serotonin agonists would reduce the activity of the serotonin neurons and thus it would decrease the release of serotonin as well as dopamine. Iderberg et al. (2015) and McCreary et al. (2016) proposed NLX-112 as a new potent serotonin 5-HT1A receptor agonist in experimental rats, and Paolone et al. (2015) and Ghiglieri et al. (2016) proposed eltoprazine as a new 5-HT1A/5-HT1B receptor agonist.

The serotonergic neurons undergo eventually neurodegeneration (Kienzl et al. 1981), but degree of neurodegeneration is much less than the dopaminergic neurons in the basal ganglia in PD (Kish et al. 2008; Buddhala et al. 2015). In addition to these data, studies on PD patients have been reported using tomography. Smith et al. (2015) studied the pallidal serotonergic neurons using a positron emission tomography in PD with dyskinesia. They used a (11)C-DASB positron emission tomography (PET), a marker of the serotonin transporter availability, and (11) a C-raclopride PET. They studied 12 PD patients with dyskinesia and 12 without dyskinesia. Levels of the GP (globus pallidus) serotonin transporter binding correlated positively with the severity of dyskinesias and they concluded that the higher GP serotonergic function was associated with levodopa-induced dyskinesias in PD. Recently, Lee et al. (2015) studied 30 patients with PD without depression or dementia, and they classified their patients into three groups, one was the non-dyskinetic, one was the dyskinetic, and the other drug naïve. They noted highest binding potential ratios ((11)C-DASB/(18)F-FP-CIT) at the putamen, which indicated serotoninergic fiber innervation relative to dopaminergic fiber availability. The binding ratio was highest in the dyskinetic group, followed by the non-dyskinetic and the drug-naive PD groups. They suggested that the serotonin/dopamine transporter ratio might be a potential marker of disease progression and an indicator of risk for levodopa-induced dyskinesia in PD. Roussakis et al. (2016) made essentially the similar observations recently. Cheshire et al. (2015) obtained the brain tissue from 44 PD patients and 17 age-matched controls and measured the dopaminergic and serotonergic markers. They found marked loss of the dopaminergic transporters but not in the serotonergic transporters in the posterior putamen of the PD patients. But the serotonergic receptor densities were not different from the dyskinetic and the non-dyskinetic patients and they concluded that the serotonergic system was not a risk factor for developing dyskinesias in PD.

Although striatal cholinergic system may influence the levodopa-induced dyskinesia, still the results of animal experiments were present (Bordia et al. 2016), and a nicotine receptor α7 agonist AQW051 had no effect on the levodopa-induced dyskinesia in patients with PD (Trenkwalder et al. 2016).

The reason why the frequency of dyskinesia was lower than those in the western countries is not well known because this is an observational study. But whenever dyskinesias appeared in the patients in our study, the individual doses of levodopa were reduced, if possible, and the numbers of drug intakes were increased, so that the total daily doses of levodopa were approximately the same as those before, and the numbers of drugs other than levodopa were reduced as possible. We believe that these modifications of the anti-parkinsonian drugs might result in the lower frequency of dyskinesia in our study.
